# Radiographic differences observed following apexification vs revascularization in necrotic immature molars and incisors: a follow-up study of 18 teeth

**DOI:** 10.1007/s40368-022-00692-z

**Published:** 2022-02-07

**Authors:** C. Caleza-Jiménez, D. Ribas-Pérez, M. Biedma-Perea, B. Solano-Mendoza, A. Mendoza-Mendoza

**Affiliations:** Department of Pediatric Dentistry, Faculty of Dentistry, University of Seville, C/ Avicena S/N, 41009 Seville, Spain

**Keywords:** Pulp treatment, Immature teeth, Necrosis

## Abstract

**Purpose:**

To evaluate the effectiveness of apexification versus revascularization in the treatment of necrotic immature teeth and determine which strategy affords the greatest radiological success rate.

**Methods:**

An analysis was made of 18 teeth subjected to mineral trioxide aggregate (MTA) apical plugging and regenerative endodontic treatment, assessing healing of the apical lesions and the changes in root dimensions.

**Results:**

Significantly greater root growth was observed with revascularization in terms of the percentage change in length (12.75% at 6 months) and dentin thickness (34.57% at 6 months) (*p* < 0.05). There were no significant differences between the two treatments in terms of the apical healing scores after 6 months of follow-up (*p* > 0.05).

**Conclusion:**

Apexification with an MTA apical plug and pulp regeneration are reliable treatments for non-vital immature teeth. The radiographic outcomes are comparable between the immature teeth subjected to MTA apexification versus those subjected to revascularization. The results of the present study indicate a greater increase in root length and width with regenerative endodontic treatment.

## Introduction

Pulp tissue alterations in immature permanent teeth can lead to loss of pulp vitality and also directly affect root development, resulting in short roots with very thin walls. This in turn increases the risk of fracture and complicates conventional root canal treatment (Guerrero et al. 2018).

Among the different treatment options in such situations, mention must be made of apexification, which induces the formation of a calcified apical barrier in an incompletely formed root in which pulp necrosis has been diagnosed (Nicoloso 2017).

Apexification may require one or more monthly patient visits to place calcium hydroxide in the root canal and eliminate intracanal infection, thus stimulating calcification and resulting in apical sealing, allowing filling to be performed with conventional techniques using guttapercha and sealing agent (Shabahang 2013). A problem with calcium hydroxide is that it can alter the mechanical properties of dentin and cause the treated tooth to be more vulnerable to root fracture (Andreasen et al. 2002). In addition, the walls of the immature permanent tooth canal remain thin and short, since formation of the hard tissue barrier is limited to the apical zone, without elongation or maturation of the root, which likewise increases the risk of root fracture (Duggal et al. 2017). Finally, calcium hydroxide apexification requires frequent replacement of the product and a long treatment period. The duration of therapy varies according to factors such as the age of the patient, the presence of periradicular radiolucent zones, and the apical width (Shabahang 2013). The treatment times for the formation of the apical barrier range between 3 and 24 months, with an average of 12 months (Ghosh et al. 2014). The time elapsed from the first patient visit to the end of treatment varies due to the multiple visits required, which complicates continuous patient monitoring and increases the vulnerability of coronal restoration, with a risk of canal reinfection.

The traditional use of calcium hydroxide for apexification is gradually replaced by the use of mineral trioxide aggregate (MTA) as a single-step technique (Purra et al. 2016) allowing shorter duration of treatment and improved patient compliance (Yadav et al. 2015). Mineral trioxide aggregate can be placed as an apical plug following previous intracanal applications of calcium hydroxide to disinfect the canal (Pace et al. 2014), and may even be used as a canal filling material itself (Chang et al. 2013). Mineral trioxide aggregate offers good biocompatibility, as evidenced by studies which have shown that MTA extrusion does not induce an adverse host response or affect periapical healing (Chang et al. 2013). Mineral trioxide aggregate also offers good sealing capacity and remains stable in a moist environment (Park and Ahn 2014). A limitation of the apical barrier technique with MTA is that it does not allow continuous root development and does not improve the crown/root proportion (Yadav et al. 2015). Short and thin roots persist in an immature permanent tooth, and MTA neither strengthens nor reinforces the tooth. As a result, the use of MTA to form an apical barrier is also associated with a future risk of root fracture, although the risk may be lower than with calcium hydroxide treatment (Evren et al. 2016). The high cost of MTA also limits its use as an apexification material (Tanalp et al. 2012).

Another strategy for the treatment of open teeth is revascularization. This is an alternative regenerative technique that allows the development of the roots and the apposition of hard tissue within the root canal (Nazzal and Duggal 2017). The concept of revascularization is based on the principle that vital stem cells are capable of surviving pulp necrosis and can differentiate into secondary odontoblasts and contribute to the conformation of root tissue (Plascencia et al. 2016). Regenerative endodontic treatment is based on the trio of tissue engineering of stem cells, growth factors and a protein scaffold such as platelet-rich plasma (PRP), within the root canal, to allow stem cell repopulation, pulp tissue regeneration, and continuation of root development with apical sealing (Nagy et al. 2014; Saoud et al. 2016). The main advantage of regenerative procedures is the continuous development of the roots, which is not observed when apexification techniques are used (Hameed et al. 2019). The disadvantages of the regenerative procedures are a greater number of patient appointments and longer treatment times compared with the MTA apical barrier technique (Staffoli et al. 2019). Moreover, not all studies have reported favorable outcomes in terms of continuous root development, and a number of authors have described a lack of increase in root length or wall thickness, and a lack of apical sealing (McTigue et al. 2013; Akcay et al. 2014). Some studies have reported that adequate intracanal bleeding is not induced—such bleeding being considered crucial for the successful delivery of stem cells, growth factors and scaffolding in root canals, and for tissue regeneration (Staffoli et al. 2019). In turn, discoloration is a common problem of regenerative endodontic procedures and may be difficult to manage (Akcay et al. 2014). Triple-antibiotic pastes containing minocycline, as well as gray and white MTA, can cause crown discoloration (Dabbagh et al. 2012). As a result, some studies have replaced minocycline with clindamycin or amoxicillin, with similar efficacy (Ray et al. 2016; Kahler et al. 2014; Alagl et al. 2017).

Revascularization is associated to significantly greater increases in root length and thickness compared with apexification using calcium hydroxide or the MTA apical barrier technique, which allow apical sealing of the treated tooth but not development of the root or—even more importantly—thickening of its root walls. In this context, revascularization affords excellent tooth-in-mouth preservation rates (Nagy et al. 2014; Jeeruphan et al. 2012; Kumar et al. 2014; Elfrink et al. 2021). Other authors (Silujjai and Linsuwanont 2017; Alobaid et al. 2014) have found revascularization to be no better than other apexification techniques in terms of the clinical or radiographic results obtained. More studies are needed, involving larger sample sizes and follow-up periods of several years to confirm whether regenerative therapy is the best option based on its clinical and biological outcomes.

The present clinical study was carried out to evaluate the effectiveness of endodontic apexification and apexogenesis treatments of permanent teeth with an open apex and to determine which strategy affords the highest radiological success rate.

## Materials and methods

### Data compilation

We collected the data corresponding to the patients treated in the Faculty of Dentistry (University of Seville, Spain) between January 2010 and December 2018. The patients were between 7 and 10 years of age and had permanent immature necrotic teeth that had been subjected to apexification and revascularization treatment. Cases identified that met the inclusion/exclusion criteria were assigned a research subject number and data was extracted from their clinical record (Table 1). Data collected included patient age and sex, tooth type and location, etiology of the disorder, clinical procedures, and follow-up time. The sample selected based on our inclusion/exclusion criteria was 18 teeth. The cases were randomized to two groups of nine patients each: Apical MTA apical plugging or regenerative endodontic treatment protocol.

**Table 1:** Inclusion/exclusion criteria

Inclusion criteria	Exclusion criteria
1. Patients without systemic diseases	1. Teeth with vertical fractures
2. Preoperative radiographs showing an immature apex accompanied by apical lesions	2. Teeth with periodontal conditions
3. Radiographic follow-up during at least 6 months after the end of treatment	3. Non-restorable teeth

### Treatment protocol

All teeth had been treated by the same operator and were isolated with rubber dams. In the cases subjected to revascularization, local anesthesia without a vasoconstrictor was administered at the first visit, with preparation of an access cavity, working length instrumentation, irrigation with 1.5–2.5% sodium hypochlorite and 17% EDTA, insertion of the product within the canal using the mixture described by Banch and Trope (2004), composed of ciprofloxacin 250 mg, metronidazole 400 mg and minocycline 50 mg in a proportion of 1:1:1 and temporary restoration. On the second visit, the formation of a blood clot within the root canal was induced, followed by MTA application with an approximate thickness of 2–3 mm and composite resin restoration of the Crown. For MTA apexification, the canals were irrigated with 2.5% sodium hypochlorite and calcium hydroxide was placed as intracanal medication between visits. The MTA was placed in the canal with an overlying cotton tip. On the next visit, the root canal was filled with injectable gutapercha.

### Radiographic evaluation

The preoperative and follow-up radiographs were obtained using the standardized paralleling technique with the Rinn XCP alignment system. The follow-up radiographs were obtained seeking to reproduce the same angulation as the preoperative radiographs. The images were stored in JPEG format and transferred to Microsoft Power Point, using the static ruler and guides to calibrate and measure the length of the root, the thickness of the dentin, and the size of the periapical lesions. Images were reviewed by two independent investigators (CC and MB), and the kappa statistic (k) was calculated to assess concordance in establishing the diagnosis. The evaluation of the radiographic images was carried out on two occasions, 1 week apart, to improve the reliability of the measurements.

**Fig. 1:**
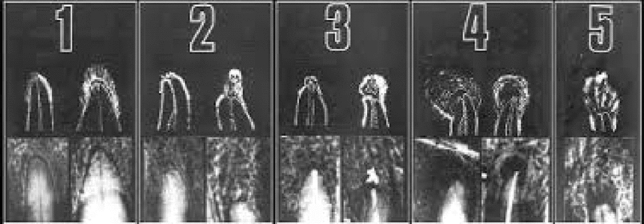
Periapical index (PAI) scores of apical periodontitis^32^ based on the reference radiographs

Apical lesions were quantified and scored from radiographic images using the periapical index (PAI) (Ørstavik et al. 1986), which represents the various stages of apical periodontitis. PAI was scored from 1 to 5, where 1 = normal periapical structure; 2 = minor changes in bone structure; 3 = changes in bone structure with some mineral loss; 4 = periodontitis with a well-defined radiolucent zone; and 5 = severe periodontitis with exacerbating characteristics as depicted in Fig. 1. Images that are most similar to the reference images were selected and their scores were recorded. The categories proposed by Friedman and Mor (2004) were modified to interpret the outcomes of treatment: healed (PAI score 1–2, with no signs of reabsorption/calcification); in process of healing (PAI score 3–4, with improvement of the score in the follow-up radiographs and no signs of reabsorption/calcification); or diseased (PAI score increasing or without change as evidenced from the follow-up radiographs after treatment, or with signs of reabsorption/calcification).

**Fig. 2:**
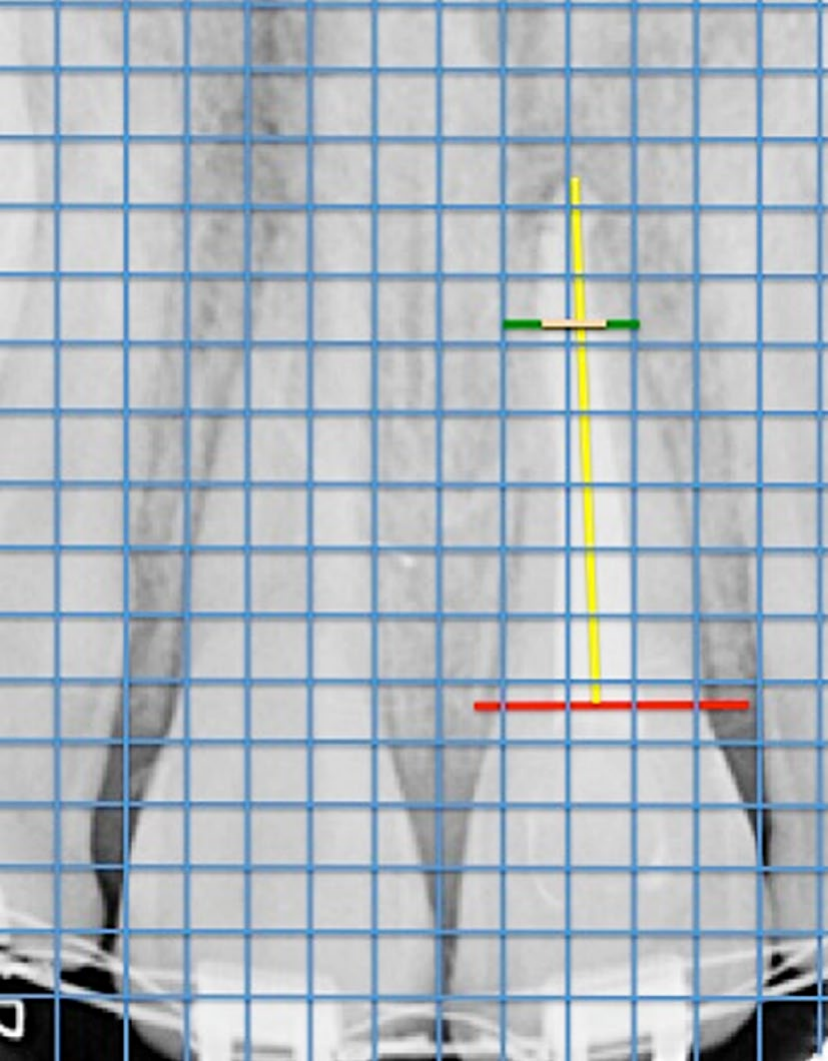
Measurement of the differences in root length and dentin wall thickness from the radiographs

Quantification of changes in the radiographic dimensions of the roots was carried out by determining variations in the length and width of the roots. Root length was measured as a straight line from the cementoenamel junction (CEJ) to the radiographic vertex of the tooth. Root canal width in turn was measured at two-thirds of the length of the preoperative root canal. The pulp space was measured at the same level, and the remaining dentin wall thickness was calculated by subtracting the pulp space from the root canal width. Differences in root length and dentin wall thickness between preoperative and follow-up radiographs were calculated as percentage changes (Fig. 2).

### Statistical analysis

The SPSS version 25 statistical package was used to process and analyze the data. The Fisher exact test was applied to analyze the differences in PAI score, with a view to exploring possible associations between the study variables based on the chi-square test.

The stages of root development were analyzed and the percentage changes in dentin wall thickness and root length were analyzed using the nonparametric Kruskal–Wallis test, since the data exhibited a nonnormal distribution. In turn, the Mann–Whitney *U* test was used to analyze differences between the groups. Statistical significance was considered for *p* < 0.05.

## Results

### Sample characteristics

During the study period 2010–2018, a total of 18 non-vital immature teeth were subjected to apexification with MTA or revascularization. The follow-up period ranged from 6 to 66 months (mean 22 ± 19, 42 months). Of the 18 teeth, 9 underwent apexification with MTA (follow-up period = 6–24 months, mean = 12.6 ± 6, 32 months), while 9 underwent revascularization (follow-up period = 12–66 months, mean = 31 ± 23, 77 months). 12 teeth were incisors that were treated with both techniques, while 6 were molars that all treated with revascularization. In some cases, we were able to obtain follow-up radiographs 3 months after the end of treatment, allowing us to assess whether there were radiological changes in that short period of time. The patients informed us whether the teeth were functional or presented symptoms. The mean age of the patient was 8 ± 1, 04 years (range 7–10). In relation to the underlying etiology, trauma accounted for 66.66% of cases and caries for 33.33% (Tables 2 and 3). Regarding the stage of preoperative root development of diseased teeth, stage 3 and 4 of Cvek were found to predominate (Tables 4 and 5).

**Table 2:** Summary of sample characteristics of apexification group

Case	Tooth	Age	Etiology	Follow-up period
1	11	10	Trauma	24 months
2	21	7	Trauma	12 months
3	11	8	Trauma	18 months
4	21	8	Trauma	12 months
5	21	9	Trauma	12 months
6	21	8	Trauma	18 months
7	21	9	Trauma	6 months
8	11	7	Trauma	6 months
9	21	8	trauma	6 months

**Table 3:** Summary of sample characteristics of revascularization group

Case	Tooth	Age	Etiology	Follow-up period
1	21	7	Trauma	12 months
2	46	9	Caries	12 months
3	36	7	Caries	60 months
4	46	9	Caries	66 months
5	11	10	Trauma	18 months
6	21	9	Trauma	30 months
7	36	8	Caries	60 months
8	36	7	Caries	12 months
**9**	46	7	Caries	12 months

**Table 4:** Summary of rooth length, root wall thickness and outcome with after apexification

Case	Tooth	Age	Etiology	Follow-up period	Preoperative Cvek’s stage of root development	Root length % change (3 months)	Dentin thickness % change (3 months)	Root length % change (6 months)	Dentin thickness % change (6 months)	Outcome
1	11	10	Trauma	24 months	4	0%	0%	0%	0%	Healed
2	21	7	Trauma	12 months	3	–	–	0%	0%	Healed
3	11	8	Trauma	18 months	3	1.13%	-6,25%	1.13%	− 6.25%	Healed
4	21	8	Trauma	12 months	3	0%	0%	0%	0%	Healed
5	21	9	Trauma	12 months	4	0%	0%	0%	0%	Healed
6	21	8	Trauma	18 months	3	0%	0%	0%	0%	Healed
7	21	9	Trauma	6 months	4	–	–	0%	− 9.09%	Healed
8	11	7	Trauma	6 months	2	–	–	1.5%	− 9.09%	Healed
9	21	8	Trauma	6 months	3	–	–	0%	− 5.88%	Healed

**Table 5:** Summary of rooth length, root wall thickness and outcome with after revascularization

Case	Tooth	Edad	Etiology	Follow-up period	Preoperative Cvek’s stage of root development	Root length % change (3 months)	Dentin thickness % change (3 months)	Root length % change (6 months)	Dentin thickness % change (6 months)	Outcome
1	21	7	Trauma	12 months	3	3.33%	13,33%	8.33%	33.33%	Healed
2	46	9	Caries	12 months	3	–	–	22.6%	5.55%	Healed
3	36	7	Caries	60 months	3	2.85%	20%	7.14%	50%	Healed
4	46	9	Caries	66 months	4	4.41%	16,66%	35.29%	33.33%	Healed
5	11	10	Trauma	18 months	4	2,35%	9,09%	5.88%	18.18%	Healed
6	21	9	Trauma	30 months	3	4.28%	11,11%	12.85%	33.33%	Healed
7	36	8	Caries	60 months	3	–	–	12.76%	37.5%	Healed
8	36	7	Caries	12 months	2	–	–	4.28%	63.63%	Healed
9	46	7	Caries	12 months	2	–	–	5.71%	36.36%	Healed

### Analysis of the dimensional changes of the roots

In analyzing postoperative radiographs after 3 and 6 months, revascularization was found to produce an increase in root width (mean = 14.03 ± 4.36% and 34.57 ± 16.62%, respectively) versus MTA apexification (− 1.25 ± 2.8% and − 3.36 ± 4.13%, respectively)—the difference being statistically significant at both time points (*p* < 0.05). The increase in root length was also significantly different between the two treatment groups (*p* < 0.05). Specifically, the percentage change in root length after three and six months was 3.44% and 12.76%, respectively, in the revascularization group versus 0.22% and 0.29% in the apexification with the MTA group (Tables 4 and 5).

The nonparametric Mann–Whitney *U* test was used since not all data exhibited a normal distribution, as evidenced by the Shapiro–Wilk test. Since *p* < 0.05 in both cases, it can be concluded that the final percentage changes in root length and dentin wall thickness differed significantly between the two treatment groups, with a greater development observed in teeth subjected to revascularization.

### Analysis of periapical healing

**Fig. 3:**
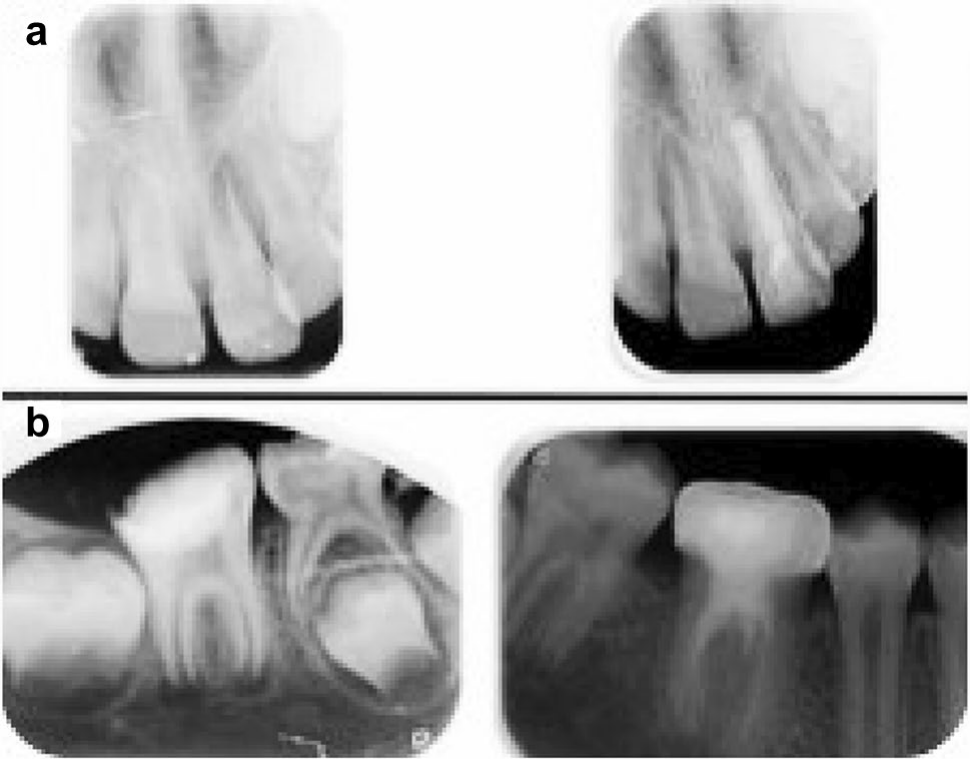
Radiographic apical healing success with MTA apical plugging (**a**) and regenerative endodontic treatment (**b**)

There were no significant differences between the two treatment groups in terms of PAI scores after 6 months of follow-up (*p* > 0.05). Both treatments exhibited good success rates (Fig. 3), with no statistically significant differences between them. Furthermore, the stages of root development did not show significant differences in the final outcome analysis.

## Discussion

The management of necrotic immature teeth has been considered an important challenge in endodontics. Regenerative approaches offer advantages over apexification, because they allow greater maturation of the root in both length and thickness thanks to regenerated vital tissue (Nazzal and Duggal 2017). Revascularization is regarded as a simple technique that can achieve pulp regeneration (Tawfik et al. 2013). The basic principle underlying pulp revascularization is filling of the empty pulp space with tissue originating in the periapical zone (Nygaard-Ostby and Hjortdal 1971), and this strategy was accepted for use in patients with avulsed immature teeth (Reynolds et al. 2009). However, such treatment was not considered possible in application to necrotic immature teeth until Iwaya et al. (2011) reported the first case of revitalization in an immature premolar with necrotic pulp tissue. Since then, the technique has gained popularity and different studies have described a number of successful treatments adopting variants of the protocol.

The present study evaluated the effectiveness of endodontic apexification and apexogenesis protocols in different permanent teeth with an open apex. Both treatments were characterized by considerable success, with no statistically significant differences in periapical healing rate between the two groups.

According to the recent systematic review of Wikströn et al. (2021), the main advantage of regenerative endodontic treatment compared to the apical plug technique is the continuous development of the root of the tooth. Many clinical studies have evidenced the possibility of root development after pulp necrosis in immature teeth (Staffoli et al. 2019). The first study to quantitatively demonstrate an increase in root length and wall thickness in teeth subjected to revascularization treatment was published in 2009; no such increase was observed in teeth subjected to MTA apexification (Bose et al. 2009). The radiographs in our study corresponding to the revascularization group and apexification with MTA group were also evaluated on a quantitative basis, and evidenced a progressive increase in root length and wall thickness in the former group that proved significantly greater than the increase observed in the apexification group.

Our findings referring to root length and thickness in the regenerative endodontic treatment group are consistent with those of other studies (Tawfik et al. 2013; Cehreli et al. 2011; Silujjai and Linsuwanont 2017; Yoshpe et al. 2021). However, Alobaid et al. (2014) found a low level of radiographic evidence of continuous root development in the revascularization group. In statistical terms, there were differences between the revascularization group and apexification with the MTA group in terms of root length and width. This coincides with practically all published case reports, case series, and retrospective and prospective cohort studies published (Staffoli et al. 2019; Nazzal and Duggal 2017; Hameed et al. 2019; Tzanetakis et al. 2021) that have shown a significantly greater increase in root length and width after revascularization versus apexification with MTA.

Continuous growth of the roots can be attributed to many mechanisms. One possible mechanism is that vital pulp cells remain at the extremity of the apical canal and may proliferate and differentiate into odontoblasts. Another possible mechanism refers to the presence of stem cells of the periodontal ligament which are able to proliferate and grow within the apical extremity through the open ápex (Banchs and Trope 2004). A third possible mechanism is based on stem cells from the apical papilla (SCAP), where instrumentation beyond the apical limit of the canal causes SCAP transplantation within the canal lumen. These stem cells may survive infection and preserve their ability to proliferate and differentiate into bone or dentin-forming cells (Gronthos et al. 2000). Lastly, a fourth possible mechanism involves the blood clot itself, since it contains growth factors, including platelet-derived growth factor, vascular endothelial growth factor, and tissue growth factors (Wang et al. 2007).

This study has some limitations, especially the simple size. It is necessary to be cautious when generalizing the results of the study. Differences in the structure of the tooth and origin of the infection in the sample may be important in terms of bacterial colonization and the composition of infected necrotic teeth. The effects of trauma and long-evolving periapical infections following pulp necrosis on the viability of the apical papilla and HERS are not clear (Jeeruphan et al. 2012). Despite the fact that fewer dimensional changes in the roots in necrotic teeth due to trauma compared to caries were observed in the revascularization group, the sample is too small. Thus, further studies are needed to better understand the true incidence of the continuous development of the root following revascularization procedures and the influence of patient- and tooth-related factors such as the cause of pulp necrosis (e.g., trauma versus caries), age, the size of the apical orifice, and the type of tooth involved. Furthermore, studies that involve longer follow-up periods could increase the incidence of clinically significant changes in the observed radiographic dimensions. Since our study was characterized by variable follow-up durations, only some of the cases could have a still greater potential for change in the root dimensions analyzed.

## Conclusions

Considering the limitations of the present study the following conclusions can be made:

- Apexification with the placement of an MTA apical plug and pulp regeneration are reliable treatments for non-vital immature teeth.
- The radiographic outcomes between the immature teeth subjected to apexification with MTA versus those subjected to revascularization were seen to be comparable.
- The results of our study indicate that regenerative endodontic treatment yields a comparatively greater increase in root length and width, however.

## Data Availability

The authors declare that there are transparency and availability of data and materials.
